# Polyphenols Inhibit *Candida albicans* and *Streptococcus mutans* Biofilm Formation

**DOI:** 10.3390/dj7020042

**Published:** 2019-04-11

**Authors:** Yosi Farkash, Mark Feldman, Isaac Ginsburg, Doron Steinberg, Miriam Shalish

**Affiliations:** 1 Biofilm Research Laboratory, Institute of Dental Sciences, Faculty of Dentistry, Hebrew University-Hadassah, P.O. Box 12065, Jerusalem 91120, Israel; markfel15@yahoo.com (M.F.); dorons@ekmd.huji.ac.il (D.S.); 2 Department of Orthodontics, School of Dental Medicine, Hebrew University-Hadassah, P.O. Box 12272, Jerusalem 91120, Israel; mshalish@hadassah.org.il; 3 Microbiology Research Laboratory, Institute of Dental Sciences, Faculty of Dentistry, Hebrew University-Hadassah, P.O. Box 12065, Jerusalem 91120, Israel; ginsburg@mail.huji.ac.il

**Keywords:** *Streptococcus mutans*, *Candida albicans*, Padma hepaten, polyphenon from green tea, polyphenols, caries

## Abstract

**Background:***Streptococcus mutans* (*S. mutans*) and *Candida albicans* (*C. albicans*) are two major contributors to dental caries. They have a symbiotic relationship, allowing them to create an enhanced biofilm. Our goal was to examine whether two natural polyphenols (Padma hepaten (PH) and a polyphenol extraction from green tea (PPFGT)) could inhibit the caries-inducing properties of *S. mutans* and *C. albicans*. **Methods:** Co-species biofilms of *S. mutans* and *C. albicans* were grown in the presence of PH and PPFGT. Biofilm formation was tested spectrophotometrically. Exopolysaccharides (EPS) secretion was quantified using confocal scanning laser microscopy. Biofilm development was also tested on orthodontic surfaces (Essix) to assess biofilm inhibition ability on such an orthodontic appliance. **Results:** PPFGT and PH dose-dependently inhibited biofilm formation without affecting the planktonic growth. We found a significant reduction in biofilm total biomass using 0.625 mg/mL PPFGT and 0.16 mg/mL PH. A concentration of 0.31 mg/mL PPFGT and 0.16 mg/mL PH inhibited the total cell growth by 54% and EPS secretion by 81%. A reduction in biofilm formation and EPS secretion was also observed on orthodontic PVC surfaces. **Conclusions:** The polyphenolic extractions PPFGT and PH have an inhibitory effect on *S. mutans* and *C. albicans* biofilm formation and EPS secretion.

## 1. Introduction

A biofilm is a structured community of diverse microbial cells enclosed in a self-produced extra cellular polymeric matrix that adheres to inert or living surfaces [1]. An established biofilm has a defined architecture, and provides the optimal conditions for the cells to avoid the human defense system and external assaults by antimicrobial agents. Dental biofilm is commonly recognized as the main cause of oral infections. Placement of orthodontic appliances usually hinders good oral hygiene and might cause alterations in the oral microflora [2]. These modifications could be responsible for decalcifications observed during orthodontic treatment [3]. *Streptococcus mutans* (*S. mutans*) is a facultative anaerobic gram-positive bacteria found in the oral cavity and in the dental biofilm, and is one of the numerous etiological factors of dental caries. Its virulence results from its ability to process sucrose from dietary substrates. Bacterial attachment to the surface of teeth, which is highly related to cariogenic activity, is mediated by the synthesis of exopolysaccharides (EPS) by the extracellular enzymes glucosyltransferase (GTF) and fructosyltransferase (FTF) [4]. It is also mediated by non-GTF glucan binding proteins. Moreover, sucrose metabolism by *S. mutans* mainly results in the production of lactic acid. *S. mutans* is one of the most acidogenic bacteria in the oral plaque. This acid production leads to a change in the plaque ecology, enhances the presence of acid-producing and acid-tolerant species, and increases the proportion of *S. mutans* in the dental biofilm [5]. The pH reduction within the plaque is responsible for enamel demineralization and confers upon the bacteria one of its most important cariogenic properties [6].

*Candida albicans (C. albicans)* is a common opportunistic pathogenic fungus in humans. It can infect mucosal membranes-causing candidiasis [7], and form biofilms on mucosal membranes as well as on implants [8]. Biofilm growth and virulence of *C. albicans* are linked to its transition from the yeast to the hyphae form, which signifies a fundamental step towards pathogenicity [9].

*C. albicans* is a colonizer of carious lesions in children and adolescents. It can ferment some dietary sugars and produce organic acids in the dental plaque, having a role in the development of caries [10,11].

The pathogenicity of *C. albicans* is attributed to production of extracellular polysaccharides (EPS), which act as adhesins, allowing attachment of *C. albicans* to host epithelial cells. In addition, the EPS are required for host recognition [12].

Studies have shown that appliances placed in the oral cavity increase the relative number of species like *Candida*, and cause substantial alterations in the oral microbiome [13,14]. Furthermore, orthodontic treatment with fixed appliances caused an elevation in *Candida* counts [15,16,17,18].

There is a symbiotic relationship between *C. albicans* and *S. mutans*. This relationship allows the microorganisms to produce an enhanced biofilm, in vitro and in vivo. Animals infected by both *C. albicans* and *S. mutans* showed higher levels of infection and microbial carriage in plaque biofilms, compared to animals infected with either species alone. Coinfection was found to augment the virulence of the biofilm, leading to the development of rampant caries [19].

Polyphenols are micronutrients in our diet that have a favorable effect as antioxidants, mostly in the oral cavity due to saliva, which improves solubilization [20], and also downstream in the stomach [21].

Second to water, the consumption of green tea, extracted from *Camellia sinensis*, is popular worldwide [22]. It contains various polyphenolic catechins—as epigallocatechin-3-gallate, which is the primary catechin, accounting for 50–80% in a cup of tea [23]. Concentrations of 250 µg/mL of this ingredient were shown to inhibit growth and biofilm formation of *S. mutans* [24]. Epicatechin-3-gallate (ECG) is the second most prevalent catechin component of green tea, and it is associated with antioxidant/anti-inflammatory properties [25].

Padma hepaten (PH) is a polyphenolic combination originated from traditional Tibetan medicine, (Padma Inc. Schwerzenbach, Switzerland). It is composed of Chebulic myrobalan, belleric myrobalan and amla fruit at a ratio of 2:1:1 [26]. Hepatic fibrosis was found to be significantly improved by PH administration [27].

In a recent study the combination of polyphenon from green tea (PPFGT) and PH was found to inhibit *C. albicans* biofilm formation and EPS secretion [28].

Inhibition of biofilm formation appears to hold promise as a natural approach to the prevention of oral diseases. The current study was designed to evaluate the suitability of polyphenols to this end. The aim of this study was to investigate whether PPFGT and PH have an inhibitory effect on *C. albicans* and *S. mutans* co-species biofilm formation and if they can prevent candidiasis, candidemia, and caries in the general population and in orthodontic patients.

## 2. Materials and Methods

### 2.1. Materials

Polyphenon 60 from green tea was obtained from Sigma-Aldrich (St. Louis, MO, USA) and Padma hepaten was obtained from Padma Inc. (Schwerzenbach, Switzerland). Both powders were dissolved and diluted in a brain‒heart infusion (BHI) to various concentrations.

### 2.2. Biofilm Growth

*C. albicans* SC5314 cells, kept in a glycerol stock at −80 °C, were thawed and incubated on BHI agar plates for 18 h at 37 °C. The cells were then diluted in BHI broth to an optical density (OD) of 0.05 at 595 nm using the Tecan GENios machine (Tecan US, Durham, NC, USA) in 96-well microtiter plates.

*S. mutans* UA159 cells were cultured in BHI at a ratio of 1:20 microorganisms to media by volume, and 2% sucrose was added to each well. The growth medium was supplemented with different concentrations of the polyphenol-rich PPFGT and PH diluted in BHI. *C. albicans* and *S. mutans* without PPFGT and PH served as a control. The mixed biofilms were developed for 48 h at 37 °C in the presence of 5% CO_2_ in 96-well plastic plates without a saliva coating.

### 2.3. Biomass of Biofilms

After incubation, the biofilms were washed gently with sterile phosphate-buffered saline (PBS). Next the biofilms were stained with Crystal Violet (CV) for 30 min; then the CV was washed away and the biofilms were washed three times with PBS. The remaining color was extracted using 33% acetic acid. The optical density (OD) was measured spectrophotometrically at 595 nm using the Tecan GENios machine (Tecan US, Durham, NC, USA) [29].

### 2.4. Confocal Laser Scanning Microscopy (CLSM)

Confocal laser scanning microscopy was used to quantify the biomass of *C. albicans*, *S. mutans*, and their EPS, and to visualize the structure and depth of the biofilms. The biofilm was prepared as described above but instead of using wild-type *C. albicans* SC5314, we used *C. albicans* SC5314 carrying the green fluorescence protein (GFP) reporter gene (*C. albicans*–GFP), kindly provided by Judith Berman (Tel Aviv University, Israel). In order to label *S. mutans* EPS, 1 mM Alexa Fluor 555-labeled dextran conjugate (10,000 MW, Molecular Probes Inc., Eugene, OR, USA) was added to the medium prior to biofilm formation. Forty-eight-hour biofilms developed in the presence of PPFGT and PH at concentrations inhibiting the biofilm growth by 50% (MBIC50) were rinsed carefully with PBS and incubated for 45 min with concanavalin A-Alexa Fluor 647 conjugate (ConA; 25 mg/mL) (Invitrogen, Carsbad, CA, USA). Con A (excitation wavelength 650 nm, emission at 668 nm) binds to the mannose and glucose residues of the EPS of fungal cell wall in a selective manner [30]. Next, *S. mutans* cells in the co-species biofilms were labeled by an immunofluorescence technique [31]. After fungal EPS staining, the biofilms were washed carefully with PBS and fixed in 4% formaldehyde solution for 1 h at room temperature. Next, the biofilms were incubated for 1 h in PBS supplement with 1% bovine serum albumin (BSA), following 1.5 h incubation with a rabbit anti-*Streptococcus mutans* polyclonal antibody (1:500; Abcam, Cambridge, UK) in PBS containing 1% BSA, followed by 1 h of incubation with Alexa fluor 405-conjugated goat anti-rabbit IgG H&L antibody (1:500; Abcam) in PBS- with 1% BSA. The stained EPS and microorganisms were identified using Zeiss LSM 510 CLS microscope (Carl Zeiss, Oberkochen, Germany). Three-dimensional images of the microbes and the EPS distribution in the biofilms were constructed using Zen2009 software (Carl Zeiss). At least three random microscopically fields were selected and analyzed. The amount of the microbial species and the individual EPS production by *S. mutans* and *C. albicans* in each sample were computed as a color-appropriated fluorescence intensity, using ImageJ v3.91 software (NIH, Bethesda, MD, USA) (http://rsb.info.nih.gov/ij). The data is displayed as the amount of fungal and bacterial cells and the individual EPS production by *C. albicans* and *S. mutans* cells in each layer of the biofilm (10 µm). The percentage of the total EPS production and the total biomass in mixed biofilms formed in the presence of polyphenols were calculated as the integral of the curve and compared to the control.

### 2.5. Morphology of the Biofilm

The biofilm’s morphology was visualized as described previously [32]. After washing, the biofilm was fixed in 4% formaldehyde for 1 h at room temperature. The morphology of the cells in the biofilm formed in the presence of PH and PPFGT was visualized using an analytical Quanta 200 Environmental High-Resolution Scanning Electron Microscope (FEI, Eindhoven, The Netherlands) at ×200–×10,000 magnification. At least three random fields were observed and analyzed.

In this experiment the biofilm was grown on poly-vinyl chloride (PVC) orthodontic surfaces (Essix) to examine whether biofilm inhibition can also occur on orthodontic surfaces.

### 2.6. Statistical Analysis

Statistical analysis was performed using a Kolmogorov‒Smirnov test for normal distribution and one-way ANOVA with a significance level of *p* < 0.05.

## 3. Results

**Figure 1:**
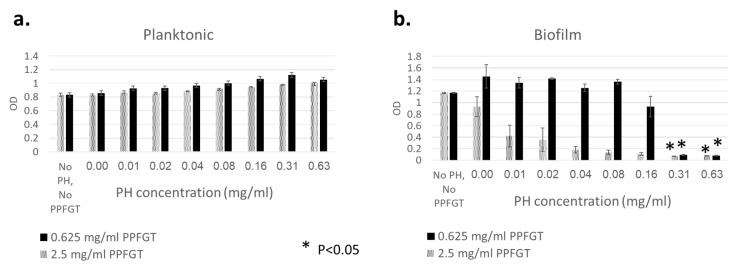
Total biomass of planktonic and biofilm cells of *C. albicans* and *S. mutans* stained with crystal violet. (**a**) OD of planktonic cells with increasing concentrations of PH. (**b**) OD of biofilm with increasing concentrations of PH. Error bars indicate the standard deviation (*n* = 96).

Figure 1a shows that there was no inhibition growth of *S. mutans* and *C. albicans* planktonic cells, regardless of the concentration of PH and PPFGT used. On the other hand, Figure 1b shows that increasing concentrations of PH inhibited the biofilm growth of *S. mutans* and *C. albicans* in a dose-dependent manner. The addition of 2.5 mg/mL PPFGT resulted in a stronger inhibition than that obtained using 0.625 mg/mL PPFGT, suggesting a combined effect of the two polyphenols.

**Figure 2:**
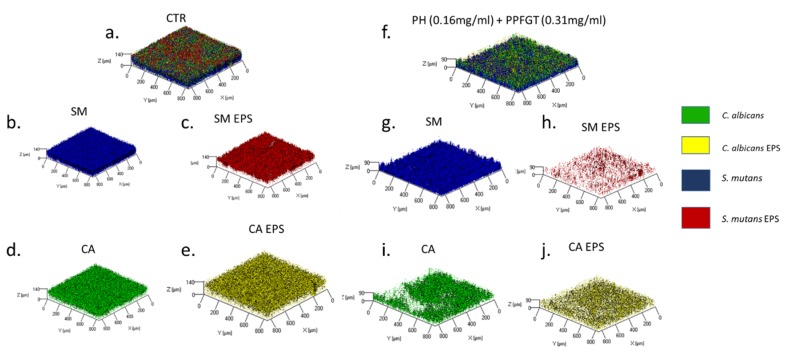
Biofilm 3D reconstruction of confocal laser scanning microscopy. Computerized 3D reconstruction (X, Y, and Z axis) of the biofilm layers (in µm), recorded using CLSM and generated by the zen2009 software. (**a**) Control *S. mutans* and *C. albicans* cells and EPS. (**b**) *S. mutans* cells only. (**c**) *S. mutans* EPS only. (**d**) Candida cells only. (**e**) Candida EPS only. (**f**) Treated *S. mutans* and *C. albicans* biofilm. (**g**) *S. mutans* cells only. (**h**) *S. mutans* EPS only. (**i**) Candida cells only. (**j**) Candida EPS only.

The three-dimensional image of the reconstructed biofilm layers (Figure 2a–j) visualizes the strong inhibition that the combination of 0.31 mg/mL PPFGT and 0.16 mg/mL PH has on biofilm formation and EPS secretion by *C. albicans* and *S. mutans*. The treated biofilm consisted of less candida and bacterial cells and less EPS than that in the untreated control group.

**Figure 3:**
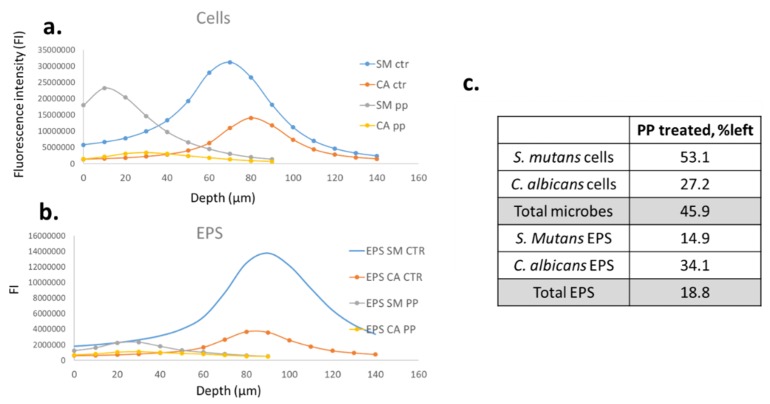
Quantification of the confocal laser scanning microscopy results. Charts a + b show the fluorescence intensity in each layer of the biofilm depth. (**a**) Cells quantification of the control and the treated groups. (**b**) EPS quantification of the control and the treated groups. (**c**) A table summing up the percentage of cells and EPS left after the various treatments relative to the control.

Figure 3a–c provides a numerical analysis of the images shown in Figure 2. The bell-shaped charts characterize the cells and EPS counts in the different layers of the biofilm. The highest counts of fluorescence were found in the middle layers of the biofilm. The numbers of the cells and EPS were reduced dramatically by the treatment with PPFGT and PH. The cell growth of *S. mutans* and *C. albicans* was inhibited by 47% and 73% and the EPS production by 85% and 66%, respectively, when the polyphenol mix was used at concentrations of 0.16 mg/mL PH and 0.31 mg/mL PPFGT.

In total, the combined cell growth of *S. mutans* and *C. albicans* was inhibited by 54% and their EPS production by 81%.

**Figure 4:**
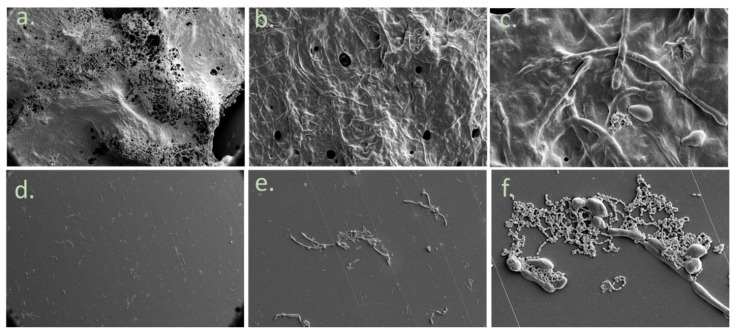
Biofilm morphology on orthodontic PVC using SEM. Morphology of the biofilm using SEM. (**a**) Control group at ×200 magnification. (**b**) at ×1000. (**c**) at ×5000. (**d**) PPFGT- & PH-treated *C. albicans* and *S. mutans* at ×200 magnification. (**e**) at ×1000. (**f**) at ×5000.

The scanning electron microscopy (SEM) results (Figure 4) indicate an inhibition of biofilm formation in the treated group (Figure 4d–f) in comparison to the control group (Figure 4a–c) tested on PVC orthodontic surfaces. The treated group contains less EPS (seen in the pictures as the blurry substance between the cells) and fewer *C. albicans* and *S. mutans* cells. In addition, the PVC surface looks much cleaner in this group.

Additional controls are presented in Appendix A.

## 4. Discussion

*S. mutans* is commonly regarded as the main cause for dental caries. *C. albicans* is often found together with *S. mutans* in plaque-biofilms from children with early childhood caries (ECC) [19]. The presence of *C. albicans* enhances EPS production, so that co-species biofilms accumulate more biomass and harbor more *S. mutans* cells compared with single-species biofilms. Co-infected animals present higher levels of infection and elevated microbial carriage in plaque biofilms compared to animals infected with one species only. Co-infection synergistically increased biofilm virulence, leading to an aggressive onset of the disease and to rampant caries [19]. Furthermore, the results of a recent study revealed that in ECC, the presence of oral *C. albicans* was related to a highly acidogenic and acid-tolerant bacterial community, with an increased amount of plaque *Streptococci* (especially *S. mutans*) [33]. *S. mutans* EPS were also found to protect *C. albicans* from the antifungal drug fluconazole [34].

The results shown in the present study demonstrate that PH and PPFGT have the capacity to inhibit the growth of *S. mutans* and *C. albicans* mixed biofilm at two important levels, the reduction of the number of the cells and by diminishing the EPS production. This effect is a specific anti-biofilm effect since at the tested concentrations of PH and PPFGT did not kill the planktonic cells.

PPFGT was studied in the past in relation to its ability to inhibit biofilm growth. However, to the best of our knowledge, we are the first to examine PH’s possible future use in the prevention of mixed biofilm-associated dental diseases.

Figure 1 shows that a significant inhibition of the co-species biofilm growth was observed using 0.16 mg/mL PH with 0.625 mg/mL PPFGT. The addition of a higher concentration of 2.5 mg/mL PPFGT yielded a stronger inhibition. Therefore, we may learn that the two polyphenols had a combined effect on the co-species biofilm growth: their combination yielded a stronger inhibition than that achieved by PH alone. However, in the same experiment, no inhibition of the planktonic co-species was observed. Another consideration that favors biofilm inhibition is that drugs penetrate poorly into biofilms and, without treatment directed at the biofilm, the response is poor and temporary [35].

Figure 2, Figure 3 and Figure 4 indicate that the reduction in the total mass of the biofilm is due to a reduction in both the number of the cells and the amount of the EPS produced. The virulence of *S. mutans* is based on its sucrose-dependent adhesion to the tooth surface, which is mediated by the production of EPS [36]. Therefore, EPS inhibition is a crucial step in caries prevention. Reduction in EPS may result in lesser adhesive forces between the cells of the biofilm causing lesser bio mass. One of the goals of clinical oral microbiology is to inhibit biofilm formation. By reducing biofilm formation with little effect on the vitality of planktonic cells, we may not affect the delicate microbial homeostasis in the oral cavity. Our study also demonstrates the ability of the polyphenols to inhibit the mixed biofilm formation on orthodontic surfaces. This is an important finding since the risk for candidiasis, caries, white spot lesions, and gingivitis, which are all biofilm-associated dental diseases, is greater in patients undergoing orthodontic treatment [2,37,38]. Few studies were performed in order to examine biofilm inhibition on orthodontic appliances. A significant reduction of salivary *S. mutans* in patients with fixed orthodontic appliances was found after probiotic yogurt consumption, which was in contrast to a control yogurt [39]. Another study examined *S. mutans* inhibition on fixed orthodontic appliances using a regular daily low dose of xylitol and revealed that the total bacterial counts in plaque and saliva as well as plaque acidogenicity remained unaffected [40]. Furthermore, the effect of sustained-release chlorhexidine varnish on orthodontic patients with fixed appliances was explored and a decrease in *S. mutans* levels up to three weeks after the application was revealed [41]. Despite its proven effectiveness, chlorhexidine use is only recommended for short periods due to its possible undesired side effects, which include desquamations and soreness in the oral mucosa, discoloration of the tongue, and tooth staining [42]. An additional study examined the ability of Cacao bean husk extract to inhibit *S. mutans* growth in vitro (on saliva-coated hydroxyapatite and orthodontic wires) and in vivo, and a significant antiplaque activity was found in both cases [43]. The minimum inhibitory concentration of garlic extract was found to be 32 mg/mL and 16 mg/mL for the inhibition of *S. mutans* and *C. albicans* biofilm on orthodontic wires, respectively [44].

The ability to inhibit biofilm growth by polyphenols, without killing planktonic cells, may lay the first stone to the development of new oral herbal medications, that might help combating oral diseases, without the creation of resistant microorganisms as a result. Since we have shown the significant effect that PH and PPFGT have on *C. albicans* and *S. mutans*, we hope that future studies, concerning their ability to affect other dental and non-dental pathogenic bacteria, may yield promising results. These two polyphenols may be capable of inhibiting biofilm formation on other surfaces as well, such as dental implants and catheters.

## Appendix A. Additional Controls

(percentage of biofilm remaining after polyphenol treatment)

*C. albicans* + PPFGT (1.25 mg/mL)—15%

*C. albicans* + PH (0.16 mg/mL)—20%

*C. albicans* + PH (0.16 mg/mL) & PPFGT (1.25 mg/mL)—12%

*C. albicans* EPS + PPFGT (1.25 mg/mL)—38%

*C. albicans* EPS + PH (0.16 mg/mL)—38%

*C. albicans* EPS + PH (0.16 mg/mL) & PPFGT (1.25 mg/mL)—26%

*S. mutans* + PPFGT (2.5 mg/mL)—48%

*S. mutans* + PH (0.16 mg/mL)—61%

*S. mutans* + PH (0.16 mg/mL) & PPFGT (2.5 mg/mL)—47%

*S. mutans* EPS + PPFGT (2.5 mg/mL)—5%

*S. mutans* EPS + PH (0.16 mg/mL)—12%

*S. mutans* EPS + PH (0.16 mg/mL) & PPFGT (2.5 mg/mL)—7%
